# Impact of premorbid personality on the course of a psychotic disorder: a case report

**DOI:** 10.1192/j.eurpsy.2026.12172

**Published:** 2026-07-31

**Authors:** C. Garcia Cerdan, M. Ligero Argudo, I. M. Peso Navarro, I. Rodríguez Blanco, L. M. Domínguez-Palacios, B. Bote Bonaechea, P. Salas Aranda, R. A. Soto Limo, C. Marín Lorenzo, A. I. Mitadiel Velasco

**Affiliations:** 1 Psychiatry; 2 Psychology, University Hospital of Salamanca, Salamanca, Spain

## Abstract

**Introduction:**

Psychotic disorders have a multifactorial origin, where biological predisposition, premorbid personality traits, and environmental factors such as substance use converge. The interaction of these elements influences not only the onset of the disorder but also its course, severity, and treatment response.

**Objectives:**

To analyze, through a clinical case, how premorbid personality traits and substance use may shape the development and progression of a long-standing psychotic disorder without previous treatment.

**Methods:**

A detailed analysis of a clinical case admitted to the Convalescence Unit of the University Hospital of Los Montalvos (Salamanca, Spain), complemented by psychometric assessment with the MCMI-IV inventory and a review of the relevant literature.

**Results:**

We present the case of a 29-year-old male, described since childhood as “strange,” with a marked tendency toward solitary play, experiences of marginalization and bullying, and progressive social withdrawal. During adulthood, he moved to live in a garage under precarious conditions, without internet access, with a daily routine limited to having lunch at his grandmother’s house, smoking cannabis daily, reading, and writing what he described as “an autobiographical book.”

In this context, he developed a chronic psychotic disorder characterized by persecutory and mystical-religious delusional ideation, complete lack of insight, and resistance to treatment. Manipulation of oral medication was suspected, leading to the initiation of depot antipsychotic treatment. As symptoms persisted, clozapine was introduced.

Psychometric evaluation with the MCMI-IV revealed paranoid, narcissistic, and turbulent personality traits, expressed through a hostile attitude, querulous discourse, grandiose self-image, and profound impermeability to external criticism.

**Conclusions:**

Premorbid personality, substance use, and psychosis do not operate as independent compartments but rather reinforce each other in a circular process: personality may facilitate and normalize substance use, which in turn aggravates psychotic symptoms, while psychosis further strengthens isolation and mistrust. This dynamic builds a cognitive structure in which it becomes clinically difficult to distinguish what corresponds to personality traits, substance use, or illness. Therefore, therapeutic management must be integral, incorporating personality assessment as an essential tool to understand treatment resistance and to design more effective interventions.

**Disclosure of Interest:**

None Declared

